# Implications of renin‐angiotensin‐system blocker discontinuation in acute decompensated heart failure with systolic dysfunction

**DOI:** 10.1002/clc.23260

**Published:** 2019-09-09

**Authors:** Douglas Darden, Mark H. Drazner, Wilfried Mullens, Matthias Dupont, W. H. Wilson Tang, Justin L. Grodin

**Affiliations:** ^1^ Division of Cardiology, Department of Internal Medicine University of California San Diego California; ^2^ Division of Cardiology, Department of Internal Medicine University of Texas Southwestern Medical Center Dallas Texas; ^3^ Department of Cardiology Ziekenhuis Oost‐Limburg Genk Belgium; ^4^ Department of Cardiovascular Medicine Heart and Vascular Institute, Cleveland Clinic Cleveland Ohio

**Keywords:** ACEI, acute decompensated heart failure, ARB, RASB, renal failure

## Abstract

**Background:**

Renin‐angiotensin‐system blockers (RASB) improve clinical outcomes in patients with chronic heart failure with reduced fraction; however, there remains ambiguity whether RASB therapy should be continued during the treatment of acute decompensated heart failure (ADHF).

**Hypothesis:**

In comparison to patients with RASB use, RASB discontinuation in ADHF will be associated with worsening renal function, hypotension, and adverse long‐term clinical outcomes.

**Methods:**

Patients in the Evaluation Study of Congestive Heart Failure and Pulmonary Artery Catheterization (ESCAPE) trial were separated into four groups based on RASB use at baseline and discharge: continuation (n = 316), discontinuation (n = 21), initiation (n = 42), and nonuse (n = 23). Post‐discharge outcomes were validated in an independent ADHF cohort admitted to the Cleveland Clinic (n = 253).

**Results:**

RASB discontinuation and nonuse were associated with higher serial creatinine and blood urea nitrogen levels than RASB continuation or initiation (*P* < .001 for both), but not with serial potassium and systolic blood pressure measurements. No other clinical parameter changes were significant. In comparison to RASB continuation, RASB discontinuation and nonuse was associated with ~75% increased risk of a 180‐day composite of death, transplant, or rehospitalization (HR 1.87, 95% CI 1.09‐3.20, *P* = 0.02 and HR 1.72, CI 1.04‐2.82, *P* = .03, respectively). Post‐discharge outcomes were similar in the validation cohort.

**Conclusion:**

Compared to RASB continuation, RASB discontinuation and nonuse were associated with higher baseline and serial creatinine levels during treatment for ADHF, but not with changes in SBP and potassium levels. Furthermore, RASB discontinuation and nonuse in ADHF were associated with an increased risk of adverse clinical outcomes.

**Abbreviations**
ACEI: ACE‐inhibitor
ADHF: acute decompensated heart failure
ARB: angiotensin‐receptor blocker
BUN: blood urea nitrogen
CC: Cleveland Clinic
ESCAPE: Evaluation Study of Congestive Heart Failure and Pulmonary Artery Catheterization Effectiveness
LVEF: left ventricular ejection fraction
PAC: pulmonary arterial catheterization
RASB: renin‐angiotensin‐system blockers
SBP: systolic blood pressure
WRF: worsening renal function

## 1 INTRODUCTION

Renin‐angiotensin‐system blockers (RASB) are cornerstones in the treatment of chronic heart failure with reduced left ventricular ejection fraction, leading to a reduction in morbidity and mortality.^1^, ^2^, ^3^, ^4^ However, there remains some ambiguity whether RASB should be continued during the treatment of patients with acute decompensated heart failure (ADHF) who develop hypotension or worsening renal function. These circumstances may explain in part why a portion of patients with ADHF are not discharged on RASB therapy despite that discharge RASB usage is considered a quality of care metric and is associated with reduced readmission rates.^5^, ^6^ Herein, in two cohorts of patients with hospitalized ADHF, we have categorized practice patterns of RASB use and determined whether such patterns were associated with renal function and subsequent long‐term outcomes.

## 2 METHODS

### 2.1 Study population

The Evaluation Study of Congestive Heart Failure and Pulmonary Artery Catheterization Effectiveness (ESCAPE) trial tested whether pulmonary arterial catheterization (PAC) improved outcomes in patients hospitalized with acute decompensated heart failure. Criteria for study inclusion were left ventricular ejection fraction ≤30%, symptomatic heart failure for at least 3 months prior despite ACEI and diuretic therapies, a systolic blood pressure (SBP) of ≤125 mm Hg and at least one sign and one symptom of congestion. There were 215 patients assigned to the PAC arm of the 433 randomized patients. This trial was conducted at 26 sites in the United States and Canada from 2000 through 2003. The main findings of this trial have been previously published.^7^ Each site approved the protocols and all patients signed informed consent prior to randomization. A publicly released version of the ESCAPE database was used for this analysis.

### 2.2 Cleveland Clinic Cohort

This cohort is three data sets pooled together from consecutive patients admitted to a specialized heart failure intensive care unit with ADHF at the Cleveland Clinic (CC) from 2000 to 2005, 2006 to 2007, and 2010 to 2011. The assembly and characteristics of this cohort have been previously described.^8^, ^9^ Briefly, medical records of all consecutive patients ≥18 years old patients were excluded if they had a history of complex congenital heart disease, received renal replacement therapy, necessitated mechanical circulatory support, or had pulmonary arterial hypertension not related to left heart failure (World Health Organization groups 1, 3, 4, and 5).^10^ All PACs were placed into the internal jugular vein in either the cardiac catheterization laboratory or an adjacent procedure room to the intensive care unit. All measurements were obtained at end expiration at steady state with the zeroing level to the phlebostatic axis. Clinical, demographic, imaging, and laboratory data and documented primary and secondary diagnoses were reviewed from the electronic medical record. Medical therapies for ADHF were started promptly following PAC insertion and at the clinicians' discretion following a structured hemodynamically guided drug titration protocol that included intravenous nitroprusside and diuretics.^11^ Inotrope use was based on clinical judgment. All patients were followed until December 31, 2011, with manual chart abstraction for orthotopic heart transplant, left ventricular assist device (LVAD) placement, hospital readmission, and death was performed. Death was additionally verified by Social Security Death Index.

### 2.3 Renin‐angiotensin system blocker use

Renin‐angiotensin system blockers (RASB) included ACE‐inhibitors (ACEI) or angiotensin‐receptor blockers (ARB). Their use was determined daily by on‐site research personnel in ESCAPE and by chart abstraction in the Cleveland Clinic (CC) cohort. Subjects with a ventricular ejection fraction (LVEF) >40% and in whom RASB could not be determined were excluded (n/N = 31/433 in ESCAPE and n/N = 190/443 in the CC cohort). The remaining cohort (ESCAPE, N = 402, CC, N = 253) was then categorized into one of groups based on RASB use at baseline and discharge: RASB at baseline and discharge [“continuation”], RASB only at discharge [“initiated”], RASB only at admission but not discharge [“discontinuation”], and no RASB use [“nonuse”].

### 2.4 Statistical methods

Continuous variables were expressed as median (interquartile range) and categorical variables as percent. The Kruskall‐Wallis and Pearson's Chi‐square tests were used to test differences across RASB use categories for continuous and categorical variables, respectively. The association of RASB use category and serial serum creatinine levels, blood urea nitrogen (BUN) levels, SBP measurements, and serum potassium levels were determined by a mixed effects model assuming unequal variance. Survival analyses were completed via the Kaplan‐Meier method and log‐rank test to compare cumulative incidence curves across RASB categories. Cox‐proportional hazards models were used to test the association between RASB and time to clinical outcomes after adjusting for potential confounders. The combined endpoint included death, transplant, or all‐cause rehospitalization. The proportional hazards assumption was verified by generalized linear regression of scaled Schoenfeld residuals over time. The model covariates were selected a priori because of their potential to confound the RASB use‐risk relationship. These included age, sex, systolic blood pressure amount < 140 mm Hg, serum potassium, and serum creatinine. All time‐to‐event analyses were landmarked at the time of discharge for each subject. Two‐sided *P*‐values <.05 were considered statistically significant. All statistical analyses were performed using Stata version 13.1 software (StataCorp LP, College Station, Texas).

## 3 RESULTS

### 3.1 Baseline characteristics

Baseline characteristics for the ESCAPE and CC cohorts are shown in Tables 1 and 2, respectively. In ESCAPE, the distribution of RASB use was continued, N = 316; initiated, N = 42; discontinued, N = 21; and nonuse, N = 23. In the CC cohort, the distribution was continued, N = 154; initiated, N = 30; discontinued, N = 28; and nonuse, N = 41. In both cohorts, patients who were not on RASB at discharge were older. In ESCAPE, those subjects who were continued or initiated on RASB had a lower baseline creatinine and BUN (continued: median 1.3 mg/dl and 27 mg/dl; initiated: 1.4 mg/dl and 31 mg/dl, respectively) than those with RASB discontinuation or nonuse (discontinuation: 2.1 mg/dl and 53 mg/dl and nonuse: 1.7 mg/dl and 56 mg/dl, respectively; *P* < .0001 for both). Similar findings were seen in the CC cohort. Notably, there were no significant differences among sex, systolic blood pressure, sodium, potassium, hemoglobin or hemodynamic characteristics across the four groups.

**Table 1:** Baseline characteristics of ESCAPE cohort

	Renin‐angiotensin system blocker prescription (baseline‐discharge)				
Variable^a^	Continuation (n = 316)	Initiation (n = 42)	Discontinuation (n = 21)	Nonuse (n = 23)	*P*‐value^b^
Age, y	54 (45‐65)	57 (51‐64)	69 (55‐79)	59 (53‐67)	.005
Male	234 (74.1)	29 (69.1)	15 (71.4)	19 (82.6)	.69
White	173 (54.8)	34 (81)	13 (61.9)	18 (78.3)	.002
Systolic blood pressure, mm Hg	104 (94‐116)	110 (96‐124)	100 (95‐118)	97 (90‐112)	.21
Ejection fraction	20 (15‐23)	20 (20‐25)	20 (15‐25)	20 (15‐25)	.10
*Medical History*					
Myocardial Infarction	122 (38.6)	25 (59.5)	14 (66.7)	16 (65.6)	<.001
Hypertension	155 (49.1)	14 (33.3)	9 (42.9)	9 (39.1)	.22
Diabetes mellitus	89 (28.8)	17 (42.5)	8 (38.1)	14 (60.9)	.006
*Signs and symptoms*					
Jugular venous pulse >8 cm H20	281 (91.5)	41 (97.6)	17 (85)	20 (90.9)	.37
Rales	166 (52.7)	22 (52.4)	11 (52.4)	12 (52.2)	1.00
Peripheral edema	103 (32.7)	18 (42.9)	10 (47.6)	12 (35.7)	.10
Baseline MLHFQ score	77.5 (67‐89)	73 (62‐83)	72 (69‐86)	73 (52‐98)	.37
*Laboratory Results*					
Sodium, mmol/l	137 (135‐140)	137 (135‐139)	136 (129‐139)	137 (133‐138)	.19
Potassium, mmol/l	4.1 (3.8‐4.5)	4 (3.6‐4.5)	4.1 (3.9‐4.8)	3.9 (3.7‐4.5)	.32
Creatinine, mg/dl	1.3 (1‐1.7)	1.4 (1.1‐1.8)	2.1 (1.6‐2.6)	1.7 (1.3‐2.5)	<.0001
Blood urea nitrogen, mg/dl	27 (18.7‐38)	31 (22‐46)	53 (31‐65)	56 (25‐83)	<.0001
Hemoglobin, mg/dl	12.6 (11.4‐13.7)	12.1 (11.6‐13.3)	9.5 (8.4‐10.7)	12.2 (10.5‐14.1)	.06
BNP, ng/l	452 (208‐911)	557 (240‐2077)	946 (660‐3130)	357 (318‐1297)	.86
*Baseline Medications*					
Beta blocker	198 (62.7)	25 (54.8)	14 (66.7)	15 (65.2)	.73
Spironolactone	143 (45.3)	15 (35.7)	6 (28.6)	9 (39.1)	.32
Digoxin	241 (76.6)	23 (54.8)	16 (80)	14 (60.9)	.009
Loop diuretics	306 (96.8)	38 (90.5)	20 (95.2)	23 (100)	.16
Nitrates	96 (30.6)	14 (33.3)	20 (28.6)	12 (52.2)	.19
Hydralazine	17 (5.5)	5 (12.5)	0 (0.0)	11 (47.8)	<.001
Inotropes	48 (15.7)	7 (16.7)	3 (14.3)	2 (8.7)	.83
*Discharge Medications*					
Beta blocker	189 (60)	18 (42.9)	10 (47.6)	14 (60.9)	.14
Spironolactone	177 (56.0)	29 (69.1)	2 (9.5)	8 (34.8)	<.001
Digoxin	250 (79.6)	37 (88.1)	13 (61.9)	14 (60.9)	.02
Nitrates	128 (40.6)	16 (38.1)	12 (57.1)	13 (56.5)	.22
Hydralazine	38 (2.3)	5 (12.5)	9 (42.9)	12 (52.2)	<.001
*Hemodynamic Characteristics (PAC arm)*	n = 154	n = 30	n = 28	n = 41	
Right atrial pressure, mm Hg	12 (8‐18)	11 (7‐17)	14 (9‐15)	18 (13‐20)	.90
Pulmonary artery systolic pressure, mm Hg	55 (45‐66)	55 (45–65)	50 (41‐62)	54 (48‐56)	.77
Pulmonary wedge pressure, mm Hg	25 (20‐30)	25 (18‐36)	22 (19‐26)	25 (23‐33)	.57
Cardiac output, l/min	3.8 (3.1‐4.7)	3.8 (2.7‐4.2)	3.3 (2.8‐3.3)	3.9 (3.4‐5.7)	.38
Cardiac index, l/min/m^2^	1.9 (1.6‐2.3)	1.9 (1.3‐2.2)	2.0 (1.4‐2.2)	1.9 (1.7‐2.7)	.36

Abbreviations: BNP, brain natriuretic peptide; MLHFQ, Minnesota Living with Heart Failure Questionnaire.

a Values are presented as median (interquartile range) for continuous and n(%) for categorical variables.

b *P*‐values for continuous variables by the Kruskal‐Wallis test and for categorical variables by the Pearson chi‐square test.

**Table 2:** Baseline characteristics of the Cleveland Clinic cohort

	Renin‐angiotensin system blocker prescription (baseline‐discharge)				
Variable^a^	Continuation (n = 154)	Initiation (n = 30)	Discontinuation (n = 28)	Nonuse (n = 41)	*P*‐value^b^
Age, y	58 (50‐65)	56 (51‐63)	68 (61‐76)	65 (58‐69)	<.0001
Male	125 (81.1)	21 (70)	16 (56.3)	31 (75.6)	.07
Systolic blood pressure, mm Hg	107 (97‐118)	81 (70‐97)	97 (87‐108)	103 (94‐116)	.06
Heart rate, beats/min	83 (73‐95)	81 (70‐97)	72 (65‐86)	81 (72‐97)	.14
Ejection fraction	15 (10‐20)	15 (10‐19)	18 (15‐20)	19 (15‐25)	.04
*Medical History*					
Ischemic cardiomyopathy	78 (50.7)	12 (40)	16 (57.1)	18 (43.9)	.51
Hyperlipidemia	87 (56.5)	16 (53.3)	14 (50)	23 (56.1)	.93
Diabetes mellitus	53 (34.4)	15 (50)	11 (39.3)	17 (41.5)	.041
Heart transplant	0	0	0	1 (0.84)	.51
*Laboratory Results*					
Sodium, mmol/l	137 (134‐140)	136 (133‐140)	137 (135‐139)	136 (134‐140)	.79
Potassium, mmol/l	4.2 (3.8‐4.6)	4.2 (3.8‐4.5)	4.2 (3.9‐4.5)	4.2 (3.8‐4.5)	.87
Creatinine, mg/dl	1.2 (0.9‐1.6)	1.3 (0.9‐1.6)	1.5 (1.2‐2.5)	1.7 (1.4‐2.4)	<.0001
Blood urea nitrogen, mg/dl	26 (19‐37)	26 (17‐36)	46 (25‐80)	41 (30‐68)	<.0001
BNP, ng/l	746 (475‐1206)	995 (663‐1829)	923 (584‐1539)	1115 (532‐1407)	.31
*Baseline Medications*					
Beta blocker	115 (75.2)	18 (60)	25 (89.3)	34 (82.9)	.04
Spironolactone	66 (42.9)	10 (33.3)	11 (39.3)	22 (53.7)	.36
Digoxin	91 (59.1)	13 (43.3)	10 (35.7)	13 (31.7)	.004
Loop diuretics	134 (87.0)	27 (90)	28 (100)	36 (87.8)	.25
Nitrates	97 (63.0)	17 (56.7)	20 (71.4)	27 (65.9)	.69
Hydralazine	73 (47.4)	16 (53.3)	20 (71.4)	27 (65.9)	.04
*Discharge Medications*					
Beta blocker	98 (63.6)	19 (63.3)	15 (53.6)	25 (61.0)	.79
Spironolactone	88 (57.1)	19 (63.3)	6 (21.4)	19 (46.3)	.003
Digoxin	87 (56.5)	17 (56.7)	7 (25.0)	10 (24.4)	<.0001
Nitrates	78 (51.3)	13 (43.3)	15 (53.6)	23 (56.1)	.75
Hydralazine	53 (34.4)	13 (43.3)	17 (60.7)	23 (56.1)	.01
*Hemodynamic Characteristics*					
Right atrial pressure, mm Hg	13 (9‐18)	16 (12‐18)	16 (12‐20)	16 (12‐22)	.06
Pulmonary artery systolic pressure, mm Hg	57 (47‐69)	64 (44‐68)	57 (52‐69)	58 (46‐70)	.94
Pulmonary wedge pressure (mean ± SD), mm Hg	27 (22‐32)	27 (23‐32)	27 (22–32)	25 (20–30)	.67
Cardiac output, l/min	3.4 (3‐4.1)	3.2 (2.3‐3.7)	3.4 (3‐4)	3.5 (2.7‐4.5)	.04
Cardiac index, l/min/m^2^	1.7 (1.5‐1.9)	1.6 (1.2‐1.8)	1.7 (1.5‐1.9)	1.8 (1.5‐2.1)	.04

Abbreviations: BNP, brain natriuretic peptide.

a Values are presented as median (interquartile range) for continuous and n(%) for categorical variables.

b *P*‐values for continuous variables by the Kruskal‐Wallis test and for categorical variables by the Pearson chi‐square test.

### 3.2 Clinical parameter changes during hospitalization

The changes in key clinical parameters, stratified by RASB classification, are shown in Table 3. Patients continued on RASB had a shorter length of stay compared to the other groups (median 6 days compared to 8 days in RASB initiation, 8 days in RASB discontinuation and 19 days in RASB nonuse, *P* = .02). However, diuretic efficiency measures and changes in serum sodium, hemoglobin, cardiac output or filling pressures were not significantly different among RASB groups.

**Table 3:** Clinical parameter changes throughout hospitalization in each RASB group for ESCAPE cohort

	Renin‐angiotensin system blocker prescription (baseline ‐ discharge)				
Characteristics^a^	Continuation (n = 305)	Initiation (n = 42)	Discontinuation (n = 21)	Nonuse (n = 23)	*P*‐value
Hospital days	6.0 (4 to 9)	8 (5 to 14)	8 (6 to 16)	19 (10 to 23)	.02
Change in Systolic BP, mm Hg	−4.0 (−14 to 5)	−6 (−25 to 6)	−1.5 (−20.5 to 4)	1 (0 to 15)	.22
Change in total weight, kg	−2.6 (−5.6 to −0.6)	−4.1 (−7.7 to −2.2)	−1.2 (−4 to −0.3)	−0.5 (−9.8 to 0)	.06
Weight change per day, kg	−0.9 (−5.8 to −0.4)	−0.9 (−4.3 to −0.5)	−0.7 (−1.7 to −0.2)	−1.5 (−2.5 to −0.02)	.49
Furosemide dose on admission, mg	200 (120 to 320)	160 (160 to 320)	180 (80 to 300)	280 (160 to 360)	.7
Furosemide equivalent dose on discharge, mg	120 (70 to 200)	120 (60 to 160)	120 (80 to 160)	160 (120‐240)	.5
*Laboratory Results*					
Sodium, mmol/l	−1 (−4 to 1)	−2 (−5 to 0)	0 (−4 to 7)	−1.5 (−4 to 0)	.16
Potassium, mmol/l	0.1 (−0.25 to 0.5)	0.2 (−0.3 to 0.7)	0.3 (−0.4 to 0.7)	0.45 (−0.45 to 0.9)	.99
Creatinine, mg/dl	0 (−0.1 to 0.2)	0 (−0.2 to 0.2)	0 (−0.6 to 0.3)	0.1 (0 to 0.4)	.92
Blood urea nitrogen, mg/dl	2 (−4 to 10)	0 (−5 to 10)	−2 (−18 to 22)	0 (−36 to 5.5)	.69
BNP, ng/l	−150.0 (−617 to 13)	−589.9 (−1788 to −277)	57.2 (−3335 to 522)	111.2 (−1830‐240)	.22
*Hemodynamic Characteristics*					
RAP, mm Hg	−3 (−7.5 to 0)	−4.5 (−10 to 0)	0 (−2 to 2)	−3 (−6 to 2)	.13
PCWP, mm Hg	−7 (−13 to −2)	−7 (−18 to 0)	−3.5 (−6 to −3)	−2 (−5 to −1)	.16
Cardiac output, l/min	0.7 (−0.1 to 1.4)	0.7 (0.1 to 1.1)	0.5 (0.1 to 2.8)	−0.1 (−0.8 to 1.6)	.31
Cardiac index, l/min/m^2^	0.7 (−0.1 to 1.4)	0.3 (0.1 to 0.6)	0.7 (0.3 to 1.4)	−0.1 (−0.8 to 1.6)	.07

Abbreviations: BP, blood pressure; BNP, brain natriuretic peptide; PCWP, pulmonary capillary wedge pressure; RAP, right atrial pressure.

a Values are presented as median (interquartile range) for continuous and n(%) for categorical variables.

**Figure 1:**
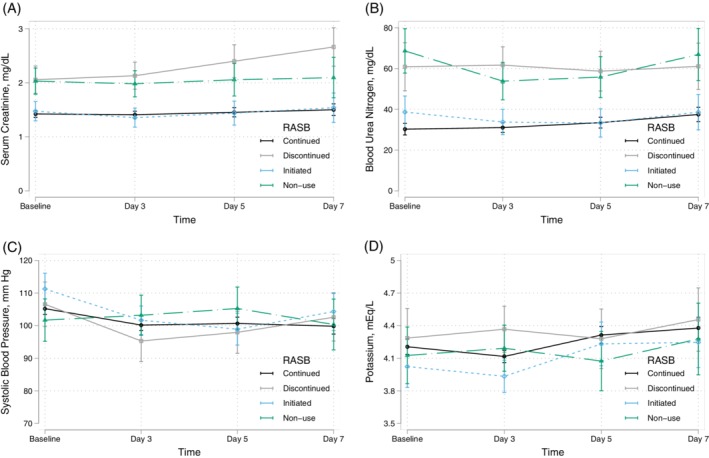
Comparison of serial changes in serum creatinine, blood urea nitrogen, systolic blood pressure, and potassium and the use of renin‐angiotensin system blockers on baseline and discharge through 7 days. Panels A, B, C, and D display the association of RASB use on serial measurements of serum creatinine, blood urea nitrogen, systolic blood pressure, and serum potassium, respectively. RASB, renin‐angiotensin system blocker

Figure 1 illustrates the results from the mixed effects models testing the association of RASB group on serial measurements of serum creatinine, BUN, systolic blood pressure, and serum potassium through 7 days. In comparison to RASB continuation, RASB discontinuation and nonuse were associated with consistently higher serial serum creatinine and BUN levels (*P* < .001 for all groups). Furthermore, RASB discontinuation was associated with rising creatinine by days 5 (*P*‐interaction = .02) and 7 (*P*‐interaction = .002). On the other hand, there was no significant difference in serial serum creatinine over time between the RASB continuation or initiation groups (*P* = .6). In comparison to RASB continuation, RASB initiation, discontinuation, and nonuse were not associated with serial systolic blood pressure measurements or potassium levels.

### 3.3 Postdischarge RASB prescription

**Figure 2:**
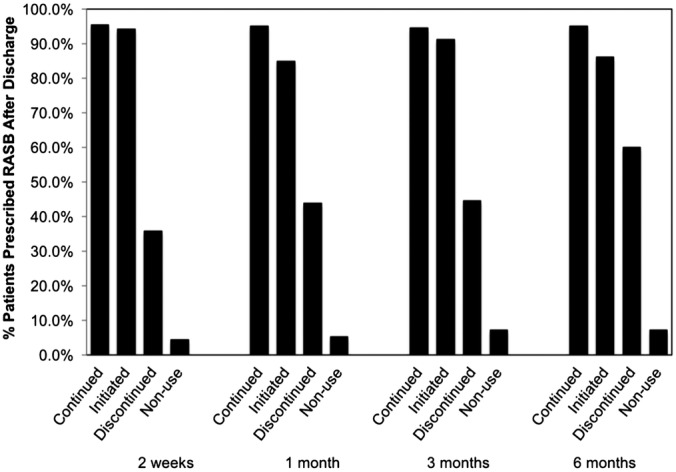
RASB use on follow‐up according to the index‐hospitalization RASB grouping. Total patients (N) at each follow‐up time point: N = 357 at 2 weeks; N = 338 at 1 month; N = 299 at 2 months; and N = 250 at 6 months. RASB, renin‐angiotensin system blocker

Figure 2 shows RASB use at 2 weeks, 1 month, 3 months, and 6 months according to the index hospitalization discharge RASB status. Those continued or initiated on RASB therapy at the time of discharge had a higher prevalence of RASB use over the next 6 months. Whereas, those with RASB discontinuation and nonuse had less RASB use over the 6 months compared to those with continued or initiated on RASB.

### 3.4 Outcomes

In ESCAPE, there were 402 patients followed for 180‐day outcomes. A total of 68 deaths occurred: 14% (n/N = 42/300) in those with RASB continuation, 14% (n/N = 6/43) in those initiated, 57% (n/N = 12/21) in those discontinued and 35% (N = 8/23) in those with nonuse (Log‐rank *P* < .001). RASB discontinuation during hospitalization was associated with a 4‐fold increase in 180‐day death (hazard ratio (HR) 4.12, 95% confidence interval (CI) 2.00‐8.47, P < 0.001), whereas RASB nonuse had a trend towards increased 180‐day death risk (HR 2.26, 95% CI 0.99‐5.15, *P* = 0.052). In the ESCAPE cohort, RASB discontinuation and nonuse during hospitalization was significantly associated with death and the composites of death, transplant, or all‐cause rehospitalization at 6 months in comparison to RASB continuation (*P* < .05 for all, Table 4). In contrast, the impact of RASB initiation was not different than RASB continuation for each outcome. Findings after multivariable adjustment were comparable. These relationships are represented in Supplemental Figure S1 with approximately 80% of patients with RASB discontinuation and nonuse reaching the composite endpoint at 6 months, compared to approximately 55% in those with RASB continuation and initiation (Log‐rank χ^2^ 19.3, *P* < .001).

**Table 4:** Cox‐proportional hazards models for the association of RASB use on baseline and discharge and long‐term outcomes in the ESCAPE and CC cohorts

Outcome	RASB Grouping	Unadjusted		Adjusted^a^	
ESCAPE 180 Day Mortality	(Baseline ‐ Discharge)	HR (95%CI)	*P*‐value	HR (95%CI)	*P*‐value
	Continuation	Reference		Reference	
	Initiation	1.11 (0.47‐2.60)	.82	1.19 (0.50‐2.84)	.69
	Discontinuation	5.60 (2.94‐10.67)	<.001	4.12 (2.00‐8.47)	<.001
	Nonuse	3.16 (1.48‐6.73)	.003	2.26 (0.99–5.15)	.052
ESCAPE 180 Day Death, Transplant or All‐Cause Rehospitalization	Continuation	Reference		Reference	
	Initiation	0.87 (0.56‐1.35)	.54	0.95 (0.61‐1.49)	.82
	Discontinuation	2.16 (1.21‐3.55)	.002	1.87 (1.09–3.20)	.02
	Nonuse	2.09 (1.30‐3.35)	.002	1.72 (1.04‐2.82)	.03
CC 180 Day Death, LVAD/ Transplant or All‐Cause Rehospitalization	Continuation	Reference		Reference	
	Initiation	0.84 (0.49‐1.45)	.54	0.79 (0.43‐1.45)	.45
	Discontinuation	2.05 (1.27‐3.3)	.003	1.85 (1.02–3.34)	.04
	Nonuse	1.42 (0.94‐2.16)	.10	1.63 (1.03–2.6)	.04

Abbreviations: LVAD, left‐ventricular assist device; RASB, renin‐angiotensin system blocker.

a Adjustment for age, male sex, systolic blood pressure < 140, potassium and baseline creatinine.

In the Cleveland Clinic cohort, there were 43 deaths, including 14.9% (n/N = 23/154) in RASB continuation, 6.7% (n/N = 2/30) in those with initiation, 35.7% (n/N = 10/28) in those with discontinuation and 19.5% (n/N = 4/21) in those with nonuse (*P* = .005). Similar trends were seen with worsened outcomes in those with RASB discontinuation and nonuse in respect to the composite endpoint of death, transplant or all‐cause hospitalization at 6 months after multivariable adjustment (HR 1.85, 95% CI 1.02 3.34, *P* = .04 and HR 1.63, 95% CI 1.03‐2.6, *P* = .04, respectively), as illustrated in Supplemental Figure S1.

## 4 DISCUSSION

We demonstrate several findings that advance our understanding of RASB use in ADHF. First, RASB discontinuation and nonuse were associated with higher serial serum creatinine and BUN during hospitalization, but not serial systolic blood pressures or serial serum potassium levels. These data suggest that renal insufficiency, rather than hypotension or potassium disturbance, may be the dominant impetus for RASB discontinuation and nonuse in patients with ADHF. Second, RASB discontinuation was not associated with subsequent diuretic efficiency or hemodynamic parameters during hospitalization. Third, RASB discontinuation and nonuse was associated with longer term RASB nonuse post discharge. Finally, RASB discontinuation and nonuse were associated with a heightened risk for long‐term adverse clinical outcomes. Altogether, these findings highlight the implications of renal function on RASB therapies and suggests RASB discontinuation and nonuse identifies a subset of patients with ADHF at higher risk of adverse events.

There remains uncertainty about the role of RASB use in patients with ADHF. The current heart failure practice guidelines caution against RASB use in patients with renal insufficiency, hypotension, or elevated potassium.^12^ However, there is little guidance regarding specifics, such as if a high baseline creatinine value contraindicates RASB use or if an increase in creatinine value necessitates discontinuation. As randomized trials demonstrating RASB benefit largely enrolled stable patients with chronic heart failure, the generalizability to the acute setting is limited.^1^, ^2^, ^3^, ^4^ Additionally, RASB use can lead to small rises in serum creatinine as reduced glomerular pressure leads to decreased glomerular filtration rate, and although this phenomenon is considered benign in patients with compensated heart failure,^13^, ^14^, ^15^, ^16^ it may create concerns among practitioners in the acute setting.

The present data are consistent with previous studies showing RASB discontinuation during ADHF is associated with worse outcomes.^17^, ^18^ In a pooled analysis of three ADHF cohorts, Vader et al similarly showed patients with RASB use at discharge had better renal function and lower risk of readmission and death.^6^ In a separate heart failure inpatient cohort, Kittleson et al showed that the discontinuation of ACEI for circulatory‐renal limitations, specifically symptomatic hypotension or progressive renal dysfunction, was associated with increased mortality.^19^ In addition to providing further evidence of the adverse RASB discontinuation or nonuse risk‐relationship, we expand upon these prior findings by providing insight that RASB discontinuation or nonuse is more likely associated with both higher serum creatinine and BUN rather than evolving hypotension. Importantly, there was a lack of association between RASB discontinuation and changing clinical and hemodynamic characteristics during hospitalization. Furthermore, no changes in renal function were observed in RASB discontinuation. Although this may suggest that RASB discontinuation may not impact the short‐term clinical course of patients with ADHF, it is unknown if RASB discontinuation may have prevented adverse hemodynamic events.

Practice patterns underlying RASB discontinuation and nonuse vary without clear supporting evidence currently to justify reasoning, particularly in patients who remain hemodynamically stable. In a cohort of 174 patients admitted with ADHF, Kane et al showed that RASB therapy was reduced or discontinued in 17.2% of patients due to acute kidney injury (56.7%), hypotension (23.3%), and hyperkalemia (10%).^20^ Admission creatinine and SBP were both associated with RASB discontinuation and nonuse. Although the rationale for RASB discontinuation or nonuse was unknown in our cohorts, the lack of an association between RASB prescription and systolic blood pressure suggests that hypotension may not be the primary impetus for RASB discontinuation. Interestingly, patients off RASB at discharge had a higher rate of hydralazine use at discharge in both cohorts, perhaps due to its substitution for RASB in the setting of increased serum creatinine. To further evaluate the safety of RASB use in those with renal insufficiency or worsening renal failure (WRF) in future studies, it will be important to concomitantly assess clinical congestion. As WRF may be observed in ADHF, adverse outcomes seem to be driven by those who remain congested.^21^, ^22^ Thus, elucidating the cause of renal insufficiency in relation to congestion may distinguish the effects of RASB therapy. For instance, in patients with WRF due to over‐diuresis, it may be appropriate to hold RASB therapy to prevent hypotension or further reductions in renal function. Conversely, a physiological rationale exists for continued RASB use in ADHF in those who remain congested with elevated creatine. RASB therapy interrupts a vicious maladaptive cycle of excessive neurohormonal activation (meant to preserve cardiac output and renal perfusion) by reducing renal pressures through the dilatation of post‐glomerular efferent arterioles and reducing cardiac filling pressures through reducing cardiac afterload, enhancing diuresis, and inhibiting angiotensin II‐induced sodium reabsorption.^15^ Whether continuing or initiating RASB therapy in those who are congested and normotensive with elevated creatinine warrants further investigation.

Although the clinical benefits of RASB use persist across the spectrum of renal function, individuals with renal insufficiency are less likely to be prescribed RASB.^23^, ^24^ While this might lead to attempts at re‐initiation of RASB in the post‐discharge outpatient setting, only a fraction of individuals with RASB discontinuation in our cohort were restarted. Although this might be reflective of the advanced nature of heart failure in our cohort likely at higher risk for more short‐term adverse clinical outcomes, it has previously been shown that those discharged without a prescription for ACEI are unlikely to be started as outpatients.^5^ Whether RASB re‐initiation post‐discharge has direct impact on clinical events is unclear but given the benefits of ongoing RASB use in both chronic heart failure and chronic renal insufficiency,^1^, ^2^, ^3^, ^4^, ^25^ the impact of RASB re‐initiation post‐discharge merits further exploration.

Our study must be interpreted in the context of several limitations inherent to its design. As a post hoc analysis, the direct impact of RASB discontinuation or nonuse in ADHF on outcomes remain uncertain. Yet, these findings highlight a high‐risk group that might benefit from further investigation. Secondly, we cannot exclude selection bias for those treated for ADHF in the context of a clinical trial. However, the generalizability of these findings is supported by comparable findings with respect to long‐term clinical outcomes in the CC cohort. Thirdly, whether there was active uptitration or downtitration of RASB during the hospitalization was unknown. While RASB uptitration has been associated with favorable clinical outcomes,^26^ dose changes in RASB were rare in our cohort and the majority (96%) that continued RASB were maintained on the same dose at discharge. As stated previously, the rationale for RASB continuation, discontinuation, initiation, or nonuse was unknown. Next, the composite outcome included all‐cause hospitalization, as opposed to heart failure or cardiovascular hospitalization, in order to validate the observations in the Cleveland Clinic cohort, which only included data on all‐cause hospitalization. Also, the multivariable models were adjusted for available risk factors to validate these findings in two cohorts. While additional clinical factors may influence the RASB risk relationship, these observations still inform the association between RASB use patterns and risk. Finally, we have equated prescription of RASB with use of RASB. Despite these limitations, detailed daily clinical data from a clinical trial allowed for serial measurements throughout hospitalization, along with validation of long‐term outcomes by a separate ADHF cohort, gives practical insight into RASB use in patients with ADHF.

## 5 CONCLUSION

In comparison to RASB continuation, RASB discontinuation and nonuse was associated with higher baseline and serial serum creatinine and BUN during treatment for ADHF, but not with changes in SBP or other key clinical parameters. Furthermore, RASB discontinuation and nonuse in ADHF were associated with an increased risk of adverse clinical outcomes. These findings further highlight the implications of RASB use at the time of discharge. Prospective study determining the cardiorenal and clinical impacts of continuing RASB therapy in the face of renal insufficiency in individuals ADHF is warranted.

## CONFLICT OF INTEREST

The authors declare no potential conflict of interest.

## Supporting information


**Supplementary Figure S1.** Kaplan–Meier Estimates of the Cumulative Incidence of the Composite Endpoints Stratified by RASB Use. The composite endpoint was death, transplant or all‐cause rehospitalization at 6 months for the ESCAPE cohort **(A)** and death, LVAD/transplant or all‐cause rehospitalization at 6 months for the Cleveland Clinic cohort **(B).**
