# Supplementary figures and images for: Implications of renin‐angiotensin‐system blocker discontinuation in acute decompensated heart failure with systolic dysfunction
Source: Clin Cardiol. 2019 Sep 9;42(10):1010–8. doi: 10.1002/clc.23260 (PMC6788475; doi:10.1002/clc.23260)

A.

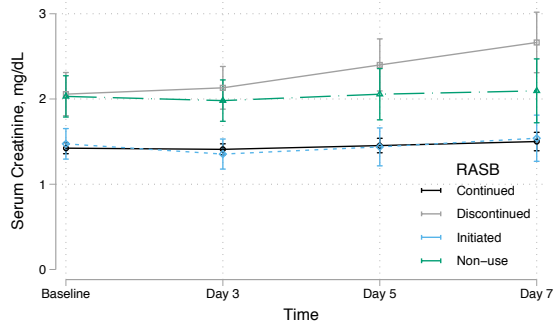

B.

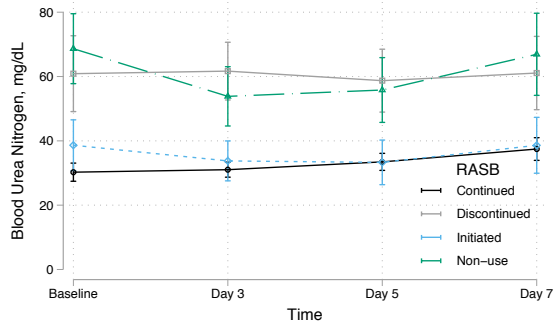

C.

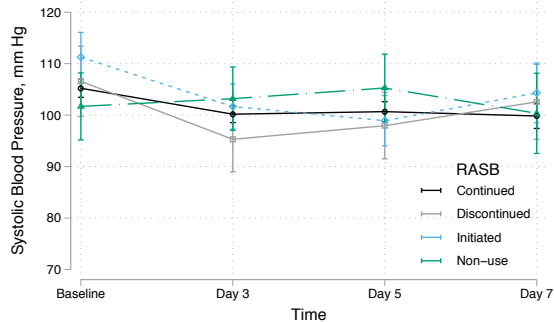

D.

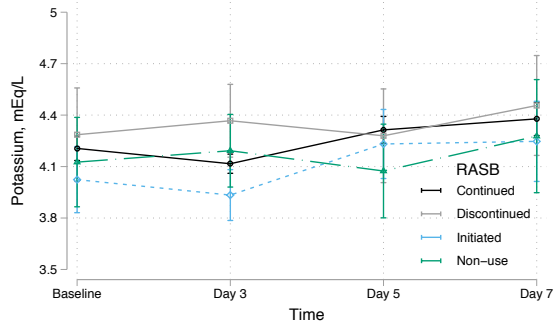

Supplement: Supplementary file 1 — Supplementary Figure S1. Kaplan–Meier Estimates of the Cumulative Incidence of the Composite Endpoints Stratified by RASB Use. The composite endpoint was death, transplant or all‐cause rehospitalization at 6 months for the ESCAPE cohort (A) and death, LVAD/transplant or all‐cause rehospitalization at 6 months for the Cleveland Clinic cohort (B). [file CLC-42-1010-s001.pdf]
